# Improved hemodynamics and cardiopulmonary function in patients with inoperable chronic thromboembolic pulmonary hypertension after balloon pulmonary angioplasty

**DOI:** 10.1186/s12931-019-1211-y

**Published:** 2019-11-08

**Authors:** Qi Jin, Qin Luo, Tao Yang, Qixian Zeng, Xue Yu, Lu Yan, Yi Zhang, Qing Zhao, Xiuping Ma, Chenhong An, Changming Xiong, Zhihui Zhao, Zhihong Liu

**Affiliations:** Center for Pulmonary Vascular Diseases, Fuwai Hospital, National Center for Cardiovascular Diseases, Chinese Academy of Medical Sciences and Peking Union Medical College, 167 Beilishi Road, Xicheng District, Beijing, 100037 China

**Keywords:** Balloon pulmonary angioplasty, Chronic thromboembolic pulmonary hypertension, Cardiopulmonary function

## Abstract

**Background:**

Balloon pulmonary angioplasty (BPA) has been demonstrated to improve cardiac function and exercise capacity in patients with inoperable chronic thromboembolic pulmonary hypertension (CTEPH), but its instant impact on cardiopulmonary function has seldom been evaluated. This study aims to determine the safety and efficacy of BPA and its immediate and lasting effects on cardiopulmonary function among CTEPH patients.

**Methods:**

From May 2018 to January 2019, patients with inoperable CTEPH who underwent BPA sessions were consecutively enrolled. Hemodynamics were measured by right heart catheterization, selective pulmonary angiography and BPA were successively conducted. Hemodynamic variables, WHO functional class (WHO-FC), 6-min walk distance (6MWD) and serum NT-proBNP were evaluated before and after BPA sessions during hospitalization. Pulmonary function testing (PFT) and cardiopulmonary exercise testing (CPET) were performed within 1–3 days pre and post BPA to evaluate the effect of BPA on cardiopulmonary function.

**Results:**

Twenty-five patients with inoperable CTEPH who underwent a total of forty BPA sessions were consecutively enrolled. A total of 183 segmental or subsegmental vessels (4.6 ± 1.9 vessels per session) in 137 segments (3.4 ± 1.6 segments per session) were dilated. No procedure-related complications occurred. Instant hemodynamics, WHO-FC, 6MWD and NT-proBNP were all significantly improved after a single BPA session. Significant improvement in cardiopulmonary function was also evident as assessed by PFT indexes (forced vital capacity, forced expiratory volume in the first second, maximal voluntary ventilation) and CPET parameters (peak work rate, peak VO_2_, oxygen uptake efficiency slope). Further analysis among ten CTEPH patients receiving multiple BPA sessions (2–4 sessions) indicated BPA resulted in lasting improvements in hemodynamics and cardiopulmonary function.

**Conclusions:**

BPA, a safe and effective approach, can bring instant improvements after a single session and lasting benefits after multiple sessions to hemodynamics and cardiopulmonary function for patients with inoperable CTEPH.

## Background

Chronic thromboembolic pulmonary hypertension (CTEPH) is a chronic and progressive disease characterized by fibrous stenosis or obliteration of pulmonary vasculature caused by organized thrombus, leading to progressive increase of pulmonary vascular resistance, right heart failure and terminal death [1–4]. The pathogenesis of CTEPH remains largely unknown, coagulation and fibrinolysis dysfunction, immune and inflammatory disorders, genetic susceptibility, defective angiogenesis and vascular remodeling have been reported to collaboratively contribute to the occurrence and development of CTEPH [5]. Although recent years have witnessed tremendous progress in medical therapies, particularly in pulmonary hypertension (PH)-targeted therapies [6], CTEPH is still a serious disease with poor prognosis with 1-, 3-, and 5-year survival rates of 90.2%, 78.4, and 64.5% respectively amongst patients with not-operated CTEPH [7].

Pulmonary endarterectomy (PEA) is recommended as the method of choice for all eligible patients with CTEPH, yet not all can benefit from this potentially curable procedure due to surgical contraindications and potential complications [4, 8, 9]. Balloon pulmonary angioplasty (BPA) was firstly reported to improve cardiac function and 6-min walk distance (6MWD) in 2001 [10], but it was accompanied with fatal reperfusion pulmonary edema and mechanical ventilation requirement, thus being left on the shelf for more than 10 years. Encouragingly, BPA has once again burst into the limelight since three gratifying reports from Japanese centers in 2012, making it as a promising alternative approach to improve hemodynamics and clinical symptoms [11–13]. Targeted therapy with or without BPA has been proposed in the CTEPH treatment algorithm for patients with non-operable CTEPH [4, 14].

Cardiopulmonary exercise testing (CPET) is a useful and non-invasive tool to objectively and safely evaluate the cardiopulmonary function and exercise capacity [15]. Our previous studies have shown CTEPH is associated with impaired ventilation efficiency and exercise capacity which are independent prognostic predictors in patients with CTEPH [16–19]. Improved exercise capacity assessed by CPET 3 months after BPA has been described [20, 21], nevertheless, the assessment was determined late and might be confounded by rehabilitation training or medical therapies. Since there are no reports regarding BPA in China and its instant impact on cardiopulmonary function among hospitalized patients with inoperable CTEPH, this study aims to firstly evaluate the safety and efficacy of BPA in our center and to assess its early effects on cardiopulmonary function in CTEPH patients.

## Methods

### Study patients

We enrolled twenty-five consecutive patients with CTEPH who underwent BPA procedures in the Center for Pulmonary Vascular Diseases, Fuwai Hospital, Chinese Academy of Medical Sciences and Peking Union Medical College from May 11, 2018 to January 4, 2019. The diagnosis criteria of CTEPH, as we previously described, was established as follows: 1) existing precapillary PH defined as mean pulmonary arterial pressure (mPAP) ≥ 25 mmHg and pulmonary arterial wedge pressure (PAWP) ≤ 15 mmHg at rest confirmed by right heart catheterization (RHC); 2) evidence of thromboembolic occlusion of the proximal or distal pulmonary vasculature detected by imaging techniques such as computed tomography, magnetic resonance imaging, ventilation/perfusion scintigraphy or pulmonary angiography; 3) the above conditions should be obtained after at least 3 months of effective anticoagulation therapy [22, 23]. Inclusion criteria for BPA procedures: all CTEPH patients who are not suitable or contraindicated for PEA after operability assessment by our multidisciplinary team including PEA surgeons, PH experts, BPA interventionists and radiologists; Those with age more than 80 years old, severe liver or kidney dysfunction, iodinated contrast agents allergy, oxygen saturation in peripheral blood < 80%, existence of foreign body such as tumor and thrombus in catheter path, severe bleeding and coagulation disorders, life-threatening hypotension and cardiac arrhythmias, and different types of tumors were excluded. Written informed consent was obtained from all patients, and this study was approved by the Fuwai Hospital Ethics Committee and conformed to the Declaration of Helsinki.

### Clinical assessment

Demographic data (age, sex and body mass index), history of deep vein thrombosis (DVT), PH targeted drugs (endothelin receptor antagonists, phosphodiesterase-5 inhibitors, prostanoids, soluble guanylate cyclase stimulators) were fully assessed after admission among all patients. World Health Organization (WHO) functional class (WHO-FC), N-terminal pro-brain natriuretic peptide (NT-proBNP) levels, oxygen saturation (SaO_2_) measured by arterial blood gas, 6MWD, transthoracic echocardiography (TTE), pulmonary function testing (PFT), chest computed tomography and pulmonary ventilation/perfusion scintigraphy before and after each BPA session within 1–3 days were well documented. General TTE parameters such as left ventricular end-diastolic diameter (LVED), right ventricular end-diastolic diameter (RVED) and tricuspid annular plane systolic excursion (TAPSE) were recorded [24]. RHC was performed through the right femoral vein after local anesthesia, hemodynamic parameters included right atrial pressure (RAP), right ventricular pressure, PAP [systolic PAP (sPAP), diastolic PAP (dPAP) and mPAP], PAWP, cardiac output (calculated by indirect Fick’s principle) and oxygen saturation (vena cava, right atrium, right ventricle and pulmonary artery). Mixed venous oxygen saturation (SvO_2_), cardiac index (CI) and pulmonary vascular resistance (PVR) were calculated according to standard formulas [20, 24].

### BPA procedures

Pulmonary angiography was performed selectively in anterior-posterior and lateral (45 degree) projections after RHC to acquire overall view about the position and degree of filling defect in combination with previous ventilation/perfusion scintigraphy. BPA was routinely performed via the right femoral vein by two interventional cardiologists (Operator: ZHZ; Assistant: TY). A 80 cm 7 Fr long sheath (Flexor® Check-Flo® Introducer; Cook Medical, Bloomington, IN, USA) was inserted into the lobar pulmonary artery to introduce a 6 Fr guiding catheter in order to prevent the guiding catheter from moving along with the beating heart as well as to facilitate the exchange of guiding catheter, wires and balloons. 6 Fr Multi-purpose (Cordis Corporation, Bridgewater, New Jersey, USA), Amplatz Left (mainly for anterior segments, especially A3; Terumo® Heartrail™ II; Terumo Corporation, Tokyo, Japan) or Judkins Right (Terumo® Heartrail™ II; Terumo Corporation, Tokyo, Japan) was selected as the guiding catheter. After introducing the guiding catheter, 2000 U unfractionated heparin was additionally infused, and oxygen was given at a flow rate of 5–8 L/min to all patients. Priority selection of target lesions was as follows: right lung > left lung, inferior lobe > superior or middle lobes, webs or bands > subtotal occlusion > chronic total occlusion > tortuous lesions. A 0.014-in. wire (Hi-Torque Pilot 50; Abbot, Santa Clara, CA, USA) was crossed to the target lesion, then appropriate balloon catheters (Mini Trek; Abbot, Santa Clara, CA, USA) were positioned over the selected lesion and inflated with iodinated contrast agents to pressures of 2–14 atm for 5–30 s. A small-sized balloon (2.0 × 20 mm) was firstly used regardless of the diameter of target vessels at initial dilation, smaller balloon may be selected to pass subtotal or total occlusion lesions, inflation pressure was dynamically adjusted according to balloon size and vessel size, and selective angiography with 1:1 mixture of saline and contrast agents was performed with deep breath holds in real time to confirm vascular filling and the presence of ruptured vessels. Dilations were repeated with unchanged or stepwise increased balloon size in case of unresponsive or poorly responsive target angiographic vessel. Hemodynamic parameters were once again measured by RHC at the end of each BPA session. A typical BPA session lasted for 1–2 h. Each session should not exceed 2000 mGy radiation exposure, 60 min fluoroscopy time, and 250 ml contrast medium. Complications including reperfusion pulmonary edema, vascular injury and hemoptysis were adjudicated by two interventional cardiologists and were well recorded.

### Cardiopulmonary exercise testing

CPET was performed according to standardized protocol as we previously described [16, 17, 25, 26]. An incremental symptom-limited exercise test was performed on an upright cycle ergometer using the COSMED Quark CPET system (COSMED, Rome, Italy). CPET was carried out prior to RHC and after BPA respectively within 3 days, and the protocol used after BPA was identical to that used before RHC. Three minutes of rest was followed by 3 min of unloaded pedaling and increased exercise using a physician-supervised, progressively increasing work rate (WR) of 5 to 30 W/min (the work rate depended on the estimated exercise capacity of each patient) to a maximum tolerance on an electromagnetically braked cycle ergometer. Patients were encouraged to exercise unless they met indications for test termination (eg, inappropriate blood pressure response, severe arrhythmia, dyspnea and leg fatigue). Gas exchange measurements were made by a metabolic cart breath by breath and averaged over 10 s intervals. Patients were continuously monitored by a standard 12-lead ECG and pulse oximetry (SpO_2_). Blood pressure was recorded using a standard cuff sphygmomanometer every 3 min. Heart rate was measured at 1-min intervals. Peak VO_2_ was defined as the highest 30s average of oxygen consumption in the last minute of exercise. Anaerobic threshold (AT) was detected by a combination of the V-slope method and ventilatory equivalents. PeakVO_2_/HR was calculated as peak VO_2_ divided by peak heart rate, and ventilatory efficiency was evaluated by minute ventilation (VE)/VCO_2_ slope and PETCO_2_@AT. VE/VCO_2_ slope was determined by linear regression analysis (VE = a VCO_2_ + b, a = slope) of VE/VCO_2_ plot obtained during exercise. Oxygen uptake efficiency slope (OUES) represented the rate of increase in VO_2_ in response to a ten-fold increase in VE during incremental exercise (VO_2_ = a log10VE + b; a = OUES) [25].

### Statistical analysis

All continuous variables were presented as mean ± SD or median (interquartile range) when appropriate, categorical variables were shown as counts or percentages. A paired t test was used to compare data from the same subject before and after BPA procedures. A two-sided *P* value < 0.05 was considered as statistically significant. Paired t test analysis was performed using SPSS version 23.0 (SPSS Inc., Chicago, IL, USA), and figures displaying changes of variables before and after BPA procedures were drawn using GraphPad Prism version 5.01 (GraphPad Software, San Diego, CA, USA).

## Results

### Study population

A total of 40 BPA sessions were performed in 25 consecutive patients (13 male, 12 female, 1.6 ± 0.9 sessions per patient) with inoperable CTEPH during the study period. Baseline clinical characteristics of CTEPH patients at the first admission, including hemodynamics measured by RHC before their initial BPA session and PH targeted therapies, were summarized in Table 1. Their mean age at baseline was 58.2 ± 9.8 years old, among these CTEPH patients, 8 (32%) had a past history of DVT. The median time from onset to admission was 3 years. 19 (76%) patients received PH targeted medications, 14 (56%) patients received monotherapy and 5 (20%) patients received combined therapy. Before the first BPA session, 12 (48%) subjects had symptoms of WHO-FC I/II while 13 (52%) were in WHO-FC III/IV. The overall CTEPH patients (*n* = 25) had a mean 6MWD of 331.8 ± 111.3 m at baseline, with a mPAP of 49.4 ± 13.4 mmHg and a mean PVR of 952.01 ± 375.38 dyn·s·cm^− 5^ .

**Table 1:** Baseline characteristics of patients with inoperable CTEPH

Variables	Values
Patients, n	25
Male/Female, n	13/12
Age, years	58.2 ± 9.8
BMI, kg/m^2^	23.6 ± 3.4
WHO-FC I/II/III/IV^a^, n	1/11/13/0
DVT, n (%)	8 (32%)
Duration from onset to admission^a^, years	3.0 (1.1–5.5)
SaO_2_^a^, %	91.3 ± 2.3
SvO_2_^a^, %	68.9 ± 5.3
mRAP^a^, mmHg	7.4 ± 3.3
mPAP^a^, mmHg	49.4 ± 13.4
PVR^a^, dyne·s·cm^−5^	952.01 ± 375.38
CI^a^, l/min/m^2^	3.18 ± 0.74
PAWP^a^, mmHg	10.2 ± 3.0
PH targeted medication^a^, n (%)	19 (76%)
Monotherapy, n (%)	14 (56%)
ERAs	2 (8%)
PDE-5is	8 (32%)
sGCs	4 (16%)
Combined therapy, n (%)	5 (20%)
ERAs+PDE-5is, n (%)	5 (20%)
6MWD^a^, m	331.8 ± 111.3
Number of BPA sessions, n	40
Number of treated segments per session, n	3.4 ± 1.6
Number of treated vessels per session, n	4.6 ± 1.9
Number of balloons per session, n	1.4 ± 0.5

*BMI* body mass index, *WHO-FC* WHO functional class, *DVT* deep vein thrombosis, *SaO*_*2*_ arterial oxygen saturation, *SvO*_*2*_ mixed venous oxygen saturation, *mRAP* mean right atrial pressure, *mPAP* mean pulmonary artery pressure, *PVR* pulmonary vascular resistance, *CI* cardiac index, *PAWP* pulmonary arterial wedge pressure, *PH* pulmonary hypertension, *ERAs* endothelin receptor antagonists, *PDE-5is* phosphodiesterase-5 inhibitors, *sGCs* soluble guanylate cyclase stimulators, *6MWD* 6-min walk distance, *BPA* balloon pulmonary angioplasty

^a^Data at the first admission

### Efficacy and safety of BPA

**Fig. 1:**
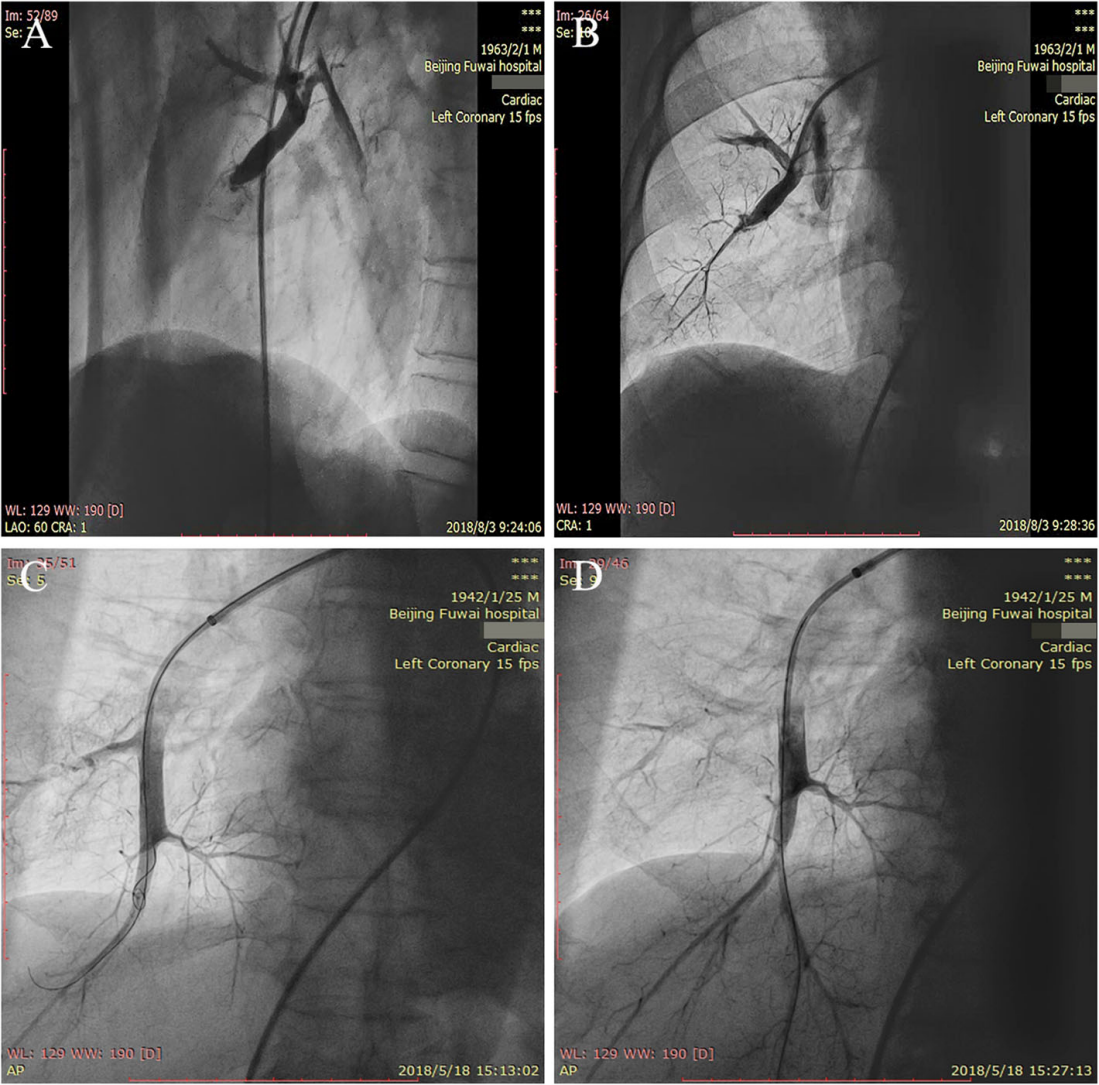
Selective pulmonary angiography in right lower lobe before and immediately after BPA. **a** Total occlusion in RA8 before BPA. **b** Improved blood flow in RA8 immediately after BPA. **c** Total occlusion in RA10 before BPA. **d** Improved blood flow in RA10 immediately after BPA

**Fig. 2:**
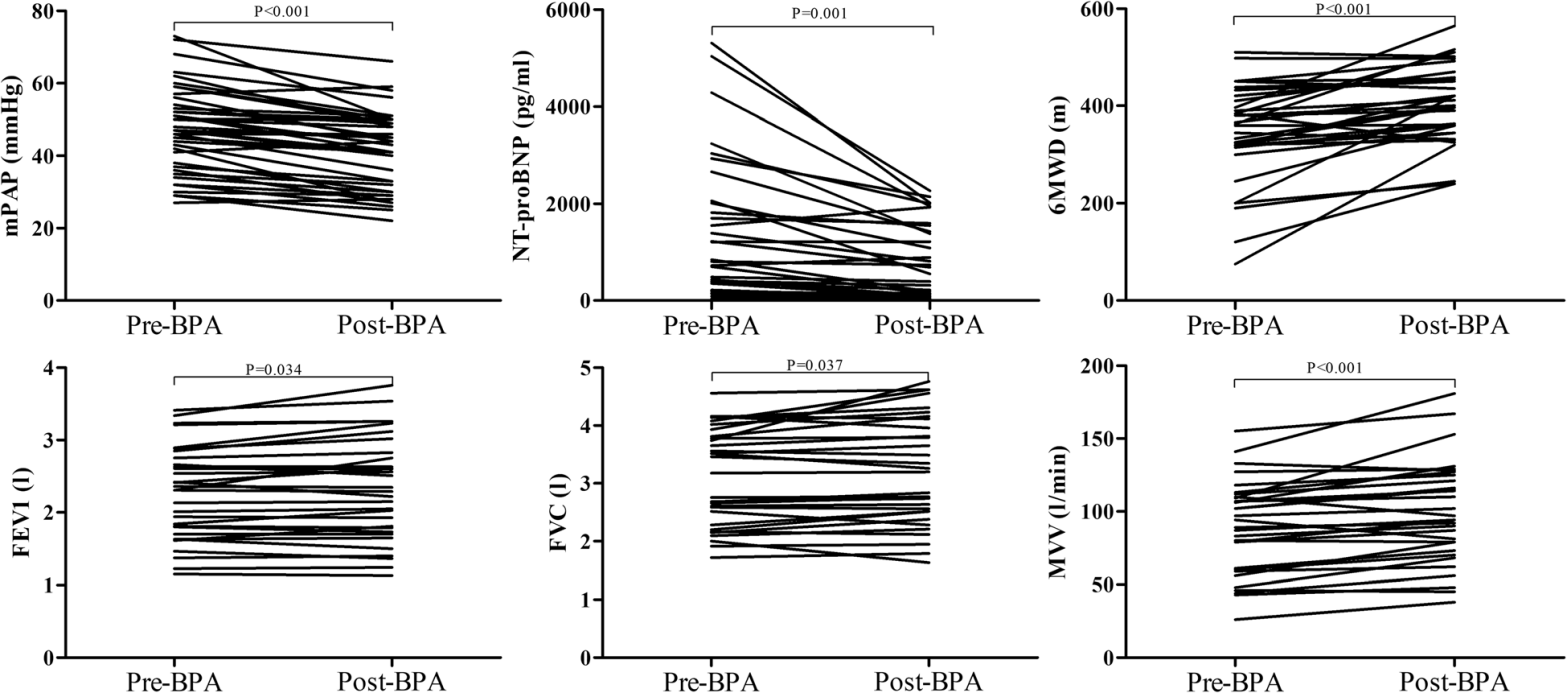
Hemodynamic and clinical parameters before and after a single BPA session

A total of 183 segmental or subsegmental vessels (4.6 ± 1.9 vessels per session) in 137 segments (3.4 ± 1.6 segments per session) were dilated among 40 BPA sessions. These vessel lesions consisted of 146 webs or bands, 17 subtotal lesions, and 20 total occlusion lesions. After selective pulmonary angiography, a total of 55 balloons (1.4 ± 0.5 per session) matched to the vessel diameters were selected, and balloon diameters and number proportion used for dilation were as follows: 1.2 mm (2%), 1.5 mm (2%), 2.0 mm (47%), 2.5 mm (13%), 3 mm (18%), 3.5 mm (16%), 4 mm (2%). Representative images and videos pre-BPA and post-BPA sessions were displayed in Fig. 1 and Additional file 1. No patients died during the follow-up period till June 1, 2019, and no complications such as pulmonary edema and hemoptysis occurred. The changes in catheter hemodynamics, echocardiographic and clinical parameters before and after BPA sessions (*n* = 40) are shown in Table 2 and Fig. 2. After BPA, CTEPH patients got immediate and significant decreases in sPAP (84.4 ± 22.8 mmHg vs 74.7 ± 21.9 mmHg, *P* < 0.001), dPAP (29.4 ± 7.8 mmHg vs 25.5 ± 7.3 mmHg, *P* < 0.001) and mPAP (47.4 ± 11.9 mmHg vs 41.5 ± 10.7 mmHg, *P* < 0.001). However, no significant differences were observed in echocardiographic parameters such as LVED, RVED and TAPSE. Average level of NT-proBNP markedly reduced from 1161.8 pg/ml to 671.2 pg/ml (*P* = 0.001). Moreover, 6MWD dramatically increased from 350.0 ± 103.4 m to 403.6 ± 81.3 m after BPA treatment. Compared with baseline, WHO-FC improved with the number of patients in I, II, III, and IV changing from 1/26/13/0 to 8/30/2/0, respectively.

**Table 2:** Hemodynamics and clinical parameters before and after a single BPA session and multiple BPA sessions

Parameters	Single BPA session (*n* = 40)			Multiple BPA sessions (*n* = 10)		
	Pre-BPA	Post-BPA	*P* value	Before-first BPA	After-last BPA	*P* value
RHC						
SvO_2_, %	69.8 ± 5.1	–		68.14 ± 4.43	72.07 ± 2.97^a^	0.019
SaO_2_, %	91.4 ± 2.1	91.9 ± 2.7	0.262	90.2 ± 1.8	91.9 ± 1.6^a^	0.01
mRAP, mmHg	7.4 ± 2.9	–		7.3 ± 3.9	8.1 ± 2.5^a^	0.54
sPAP, mmHg	84.4 ± 22.8	74.7 ± 21.9	< 0.001	93.0 ± 26.0	81.2 ± 18.7^a^	0.068
dPAP, mmHg	29.4 ± 7.8	25.5 ± 7.3	< 0.001	31.9 ± 9.0	27.8 ± 4.3^a^	0.089
mPAP, mmHg	47.4 ± 11.9	41.5 ± 10.7	< 0.001	52.6 ± 14.1	44.8 ± 8.2^a^	0.031
PVR, dyne·s·cm^−5^	886.15 ± 342.04	–		1067.0 ± 410.4	758.0 ± 212.1^a^	0.04
PAWP, mmHg	9.8 ± 3.2	–		8.1 ± 2.8	10.7 ± 3.0^a^	0.164
CI, l/min/m^2^	3.26 ± 0.78	–		3.07 ± 0.69	3.42 ± 0.70^a^	0.202
TTE						
LVED, mm	41.1 ± 4.9	42.0 ± 5.1	0.203	41.4 ± 3.7	43.7 ± 3.8	0.048
RVED, mm	31.3 ± 7.4	30.2 ± 6.4	0.142	30.5 ± 4.0	28.1 ± 5.1	0.32
TAPSE, mm	16.5 ± 4.2	17.2 ± 3.5	0.296	16.2 ± 4.2	18.3 ± 1.5	0.304
Pulmonary function testing						
FVC, l	3.11 ± 0.83	3.21 ± 0.93	0.037	3.22 ± 0.84	3.49 ± 1.01	0.01
FVC% predicted, %	90.7 ± 13.0	93.4 ± 14.5	0.041	95.8 ± 12.0	103.6 ± 11.2	0.004
FEV1, l	2.26 ± 0.64	2.32 ± 0.71	0.034	2.39 ± 0.69	2.55 ± 0.79	0.023
FEV1% predicted, %	80.8 ± 14.2	82.7 ± 16.1	0.045	87.9 ± 16.5	93.2 ± 15.4	0.038
MVV, l/min	89.4 ± 31.7	99.1 ± 34.7	< 0.001	95.2 ± 37.7	114.1 ± 36.2	0.002
MVV% predicted, %	83.2 ± 24.0	92.4 ± 25.2	< 0.001	89.0 ± 26.4	108.4 ± 26.0	0.005
NT-proBNP, pg/ml	1161.8 ± 1420.0	671.2 ± 753.0	0.001	1329.0 ± 1599.0	326.5 ± 601.0	0.022
6MWD, m	350.0 ± 103.4	403.6 ± 81.3	< 0.001	316.8 ± 120.2	443.9 ± 63.6	0.003
WHO-FC I/II/III/IV	2.3 ± 0.5	1.9 ± 0.5	< 0.001	2.7 ± 0.5	2.0 ± 0	0.001
I, n (%)	1 (2.5%)	8 (20%)		0 (0%)	0 (0%)	
II, n (%)	26 (65%)	30 (75%)		3 (30%)	10 (100%)	
III, n (%)	13 (32.5%)	2 (5%)		7 (70%)	0 (0%)	
IV, n (%)	0 (0%)	0 (0%)		0 (0%)	0 (0%)	

*BPA* balloon pulmonary angioplasty, *RHC* right heart catheterization, *SvO*_*2*_ mixed venous oxygen saturation, *SaO*_*2*_ arterial oxygen saturation, *mRAP* mean right atrial pressure, *sPAP* systolic pulmonary artery pressure, *dPAP* diastolic pulmonary artery pressure, *mPAP* mean pulmonary artery pressure, *PVR* pulmonary vascular resistance, *PAWP* pulmonary arterial wedge pressure, *CI* cardiac index, *TTE* transthoracic echocardiography, *LVED* left ventricular end-diastolic diameter, *RVED* right ventricular end-diastolic diameter, *TAPSE* tricuspid annular plane systolic excursion, *FVC* forced vital capacity, *FEV1* forced expiratory volume in the first second, *MVV* maximal voluntary ventilation, *NT-proBNP* N-terminal pro-brain natriuretic peptide, *6MWD* 6-min walk distance, *WHO-FC* WHO functional class

^a^Data at RHC before the last BPA

### Cardiopulmonary function after a single BPA session

**Fig. 3:**
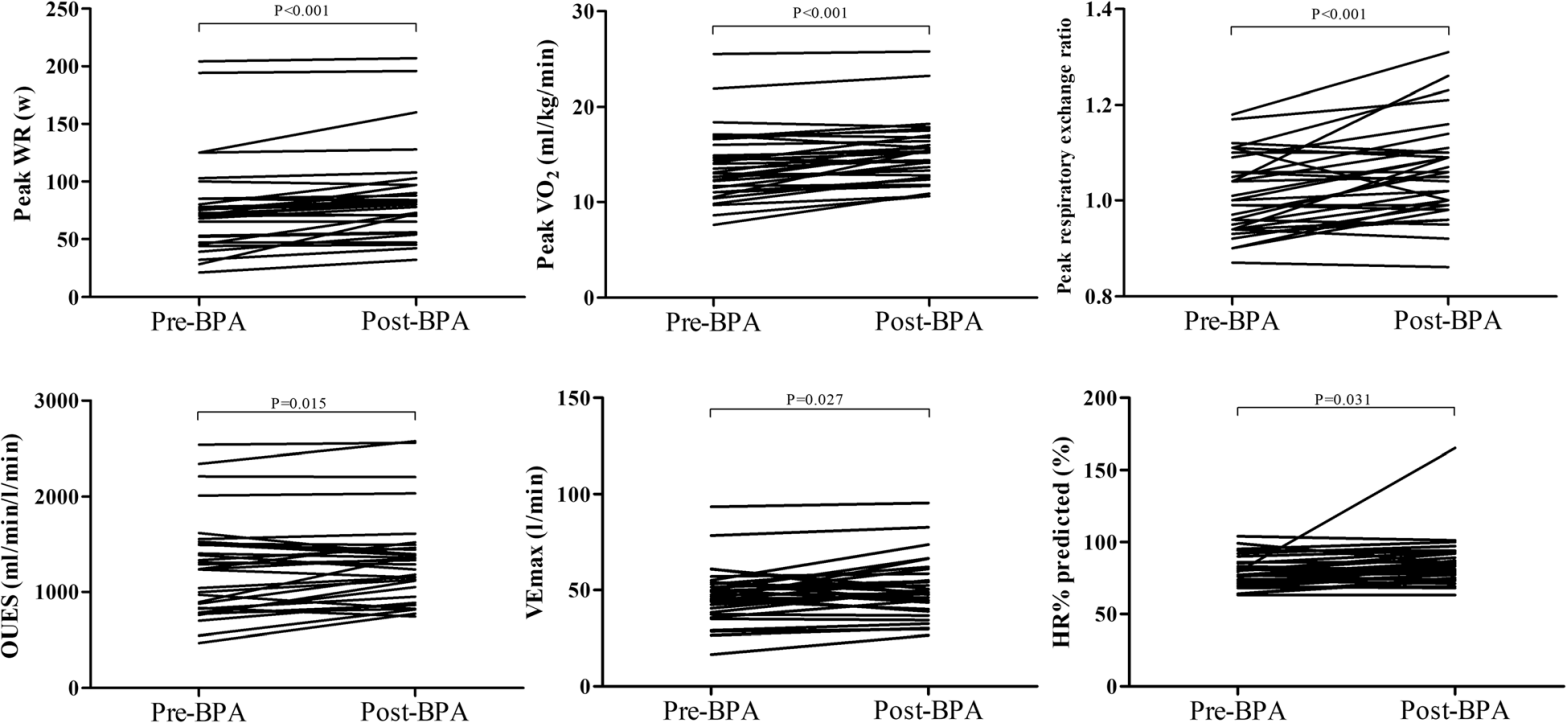
CPET parameters before and after a single BPA session

As for PFT, forced vital capacity (FVC), FVC% predicted, forced expiratory volume in the first second (FEV1), FEV1% predicted, maximal voluntary ventilation (MVV) and MVV% predicted all significantly improved post-BPA compared to those of pre-BPA (all *P* < 0.05; Fig. 2). As shown in Table 3 and Fig. 3, exercise performance as evaluated by several CPET parameters improved significantly after BPA. Compared to pre-BPA, Peak WR (78.0 ± 40.6 w vs 86.7 ± 39.8 w, *P* < 0.001), Peak VO_2_ (13.9 ± 3.7 ml/kg/min vs 15.2 ± 3.3 ml/kg/min, *P* < 0.001), Peak VO_2_% predicted (54.5 ± 16.8% vs 58.8 ± 15.3%, *P* < 0.001), peak respiratory exchange ratio (1.01 ± 0.08 vs 1.06 ± 0.10, *P* < 0.001), OUES (1250.5 ± 508.3 ml/min/l/min vs 1329.1 ± 466.6 ml/min/l/min, *P* = 0.015) and HR% predicted (78.5 ± 10.5% vs 84.9 ± 17.4%, *P* = 0.031) all significantly increased post-BPA. Similarly, notable increments were also identified in ventilation parameters VEmax (47.2 ± 14.4 l/min vs 50.7 ± 15.1 l/min, *P* = 0.027), VT@AT (1.19 ± 0.31 l vs 1.31 ± 0.34 l, *P* = 0.035) and VTmax (1.54 ± 0.38 l vs 1.67 ± 0.43 l, *P* < 0.001). HR@ AT (102.8 ± 18.3 bpm vs 108.1 ± 19.0 bpm, *P* = 0.056) and Peak VO_2_/HR% predicted (69.0 ± 17.9% vs 71.3 ± 15.3%, *P* = 0.068) before and after BPA reached a borderline statistical significance. VE/VCO_2_ slope, VE/VCO_2_ slope% predicted and Peak VE/VCO_2_ were on a downward trend, although no significant differences were found between pre-BPA and post-BPA.

**Table 3:** CPET parameters before and after a single BPA session and multiple BPA sessions

Parameters	Single BPA session (*n* = 40)			Multiple BPA sessions (*n* = 10)		
	Pre-BPA	Post-BPA	*P* value	Before-first BPA	After-last BPA	*P* value
Peak WR, w	78.0 ± 40.6	86.7 ± 39.8	< 0.001	77.3 ± 47.6	96.2 ± 40.2	0.024
AT, ml/kg/min	10.7 ± 3.2	11.7 ± 3.1	0.098	10.3 ± 3.2	11.4 ± 3.3	0.57
Peak VO_2_, ml/kg/min	13.9 ± 3.7	15.2 ± 3.3	< 0.001	13.7 ± 4.5	16.1 ± 3.2	0.025
Peak VO_2_% predicted, %	54.5 ± 16.8	58.8 ± 15.3	< 0.001	53.7 ± 18.1	63.9 ± 14.1	0.015
Peak respiratory exchange ratio	1.01 ± 0.08	1.06 ± 0.10	< 0.001	1.04 ± 0.08	1.09 ± 0.10	0.046
VE/VCO_2_ slope	49.1 ± 15.3	45.3 ± 11.4	0.09	52.3 ± 16.7	44.0 ± 10.5	0.099
VE/VCO_2_ slope% predicted, %	181.4 ± 57.0	166.7 ± 40.6	0.079	191.4 ± 62.7	160.7 ± 37.8	0.098
OUES, ml/min/l/min	1250.5 ± 508.3	1329.1 ± 466.6	0.015	1205.0 ± 576.2	1341.0 ± 382.6	0.397
VEmax, l/min	47.2 ± 14.4	50.7 ± 15.1	0.027	52.4 ± 17.1	56.8 ± 15.4	0.197
VT@AT, l	1.19 ± 0.31	1.31 ± 0.34	0.035	1.20 ± 0.37	1.31 ± 0.33	0.422
VTmax, l	1.54 ± 0.38	1.67 ± 0.43	< 0.001	1.66 ± 0.39	1.80 ± 0.45	0.051
HR@AT, bpm	102.8 ± 18.3	108.1 ± 19.0	0.056	102.3 ± 15.1	108.6 ± 14.4	0.388
HRmax, bpm	128.9 ± 19.5	132.9 ± 16.0	0.16	126.4 ± 17.0	141.8 ± 13.0	< 0.001
HR% predicted, %	78.5 ± 10.5	84.9 ± 17.4	0.031	79.7 ± 9.8	88.7 ± 7.5	< 0.001
Peak VO_2_/HR, ml/bpm	7.52 ± 2.88	7.66 ± 2.55	0.381	7.24 ± 3.37	7.73 ± 2.49	0.466
Peak VO_2_/HR% predicted, %	69.0 ± 17.9	71.3 ± 15.3	0.068	66.6 ± 18.9	71.8 ± 12.0	0.272
Peak PetCO_2_, mmHg	24.7 ± 6.3	24.6 ± 7.0	0.916	22.4 ± 5.3	24.4 ± 4.9	0.39
Peak PetO_2_, mmHg	125.0 ± 5.0	125.7 ± 4.9	0.253	126.8 ± 3.8	127.8 ± 3.8	0.454
Peak VE/VO_2_	52.3 ± 12.6	51.6 ± 10.6	0.609	60.1 ± 14.4	54.4 ± 9.6	0.359
Peak VE/VCO_2_	51.8 ± 12.9	49.6 ± 10.7	0.078	57.9 ± 13.5	50.5 ± 10.5	0.19
Peak SpO_2_, %	91.0 ± 5.1	92.4 ± 4.7	0.484	89.1 ± 4.6	90.9 ± 4.1	0.213

*Peak WR* peak work rate, *AT* anaerobic threshold, *Peak VO*_*2*_ peak oxygen consumption, *VE/VCO*_*2*_ minute ventilation/carbon dioxide production, *OUES* oxygen uptake efficiency slope, *VEmax* maximal minute ventilation, *VT@AT* tidal volume at the anaerobic threshold, *VTmax* maximum tidal volume, *HR@AT* heart rate at the anaerobic threshold, *HRmax* maximum heart rate, *Peak VO*_*2*_*/HR* peak oxygen consumption/peak heart rate, *PetCO*_*2*_ partial pressure of end-tidal carbon dioxide, *PetO*_*2*_ partial pressure of end-tidal oxygen, *VE/VO*_*2*_ minute ventilation/oxygen consumption, *SpO*_*2*_ pulse oxygen saturation

### Hemodynamics and cardiopulmonary function after multiple BPA sessions

For ten CTEPH patients receiving repeated BPA sessions (2–4 sessions), a subgroup analysis was performed. The median time interval between the first and last BPA sessions was 14.4 (9.8–29.2) weeks. As described in Table 2, SaO_2_ (90.2 ± 1.8% vs 91.9 ± 1.6%, *P* = 0.01) and SvO_2_ (68.14 ± 4.43% vs 72.07 ± 2.97%, *P* = 0.019) were both significantly increased before the last BPA session when compared with those before the first BPA session. In addition, mPAP decreased from 52.6 ± 14.1 mmHg to 44.8 ± 8.2 mmHg (*P* = 0.031, Additional file 2: Figure S1), and PVR declined from 1067.0 ± 410.4 dyne·s·cm^− 5^ to 758.0 ± 212.1 dyne·s·cm^− 5^, while CI remained unchanged. LVED measured by TTE was significantly elevated from 41.4 ± 3.7 mm to 43.7 ± 3.8 mm. In contrast to data before first-BPA, all variables of PFT as was mentioned above augmented significantly after last-BPA. Furthermore, the average level of NT-proBNP fell significantly over time from 1329.0 pg/ml to 326.5 pg/ml, while 6MWD rose to 443.9 ± 63.6 m after the last BPA session (Additional file 2: Figure S1). Seven (70%) patients in WHO-FC III prior to the first BPA session improved to WHO-FC II after the final procedure. The changes of CPET parameters before-first and after-last BPA sessions were displayed in Table 3. Compared with baseline, Peak WR, Peak VO_2_, Peak VO_2_% predicted, peak respiratory exchange ratio and HR% predicted were still obviously elevated (Additional file 2: Figure S2). HRmax during exercise increased significantly from 126.4 ± 17.0 bpm to 141.8 ± 13.0 bpm (*P* < 0.001), yet differences of VTmax between before-first BPA and after-last BPA reached a marginal significance (*P* = 0.051). Non-significant increased trends in OUES, Peak VO_2_/HR and SpO_2_ were discerned.

## Discussion

This is, to the best of our knowledge, the first study to investigate the safety and efficacy of BPA in China, as well as its instant effects on cardiopulmonary function in patients with inoperable CTEPH. The present study demonstrated BPA, as a safe and effective approach, brought instant and lasting benefits to hemodynamics and cardiopulmonary function for patients with inoperable CTEPH.

PEA is currently the only established curative treatment for patients with CTEPH [14]. Nevertheless, nearly 40% of subjects were considered inoperable because of lesion inaccessbility or possible comorbidities [4, 8], and 25–31% of CTEPH patients had residual PH after PEA surgery [27, 28]. Moreover, PEA surgery involves exquisite techniques and multidisciplinary collaboration, and can be performed only at a limited number of institutions. Riociguat, a soluble guanylate cyclase stimulator, is the only approved medical therapy indicated for patients with inoperable CTEPH based on the CHEST trials [6, 29, 30]. However, majorities of CTEPH patients cannot benefit from this drug for economic reasons in China.

BPA is an interventional technique which uses a balloon catheter to cross and dilate target lesions to relieve pulmonary vascular stenosis or obstruction. It was firstly attempted in 1983 to relieve pulmonary artery stenosis or hypoplasia, and brought significant hemodynamic improvements for children with congenital lesions [31]. The first case of BPA for CTEPH was reported by Voorburg et al. in 1988 [32]. Later in 2001, Feinstein described 18 CTEPH case series treated with BPA, showing improved hemodynamics and exercise tolerance. Unfortunately, 11 patients developed reperfusion pulmonary edema, and 3 patients required mechanical ventilation [10]. With continuous refinements from three Japanese institutions [11–13], BPA has been reported to significantly improve hemodynamics, exercise capacity and right ventricular function, with significantly lower rates of complications [33–36]. BPA is now recommended in the newly proposed CTEPH treatment algorithm for inoperable CTEPH patients at experienced centers [4], however, most of data with regard to BPA in CTEPH mainly come from Japan, Europe and USA, the safety and efficacy of BPA procedures in China have never been reported.

Our study demonstrated that BPA conferred significant improvements in instant hemodynamics as evidenced by decreased sPAP, dPAP and mPAP. However, differential structural changes in the heart evaluated by TTE were not observed after a single BPA session among our patients. In accordance with our results, Tsugu et al. [33] also noticed mPAP was significantly improved, and right ventricular basal, mid cavity and longitudinal diameters remained unchanged immediately after BPA, but left ventricular end-diastolic diameter and TAPSE obviously increased compared with baseline, the difference might be explained by relatively better right ventricular function and higher TAPSE of our patients. In order to exclude the effect of supplemental oxygen on pulmonary hemodynamics during BPA procedures, we compared catheterization hemodynamics before the first BPA and before the second BPA among patients receiving multiple BPA sessions which revealed mPAP decreased from 52.6 ± 14.1 mmHg to 44.5 ± 9.2 mmHg (*P* = 0.004) and PVR declined from 1067.0 ± 410.4 dyne·s·cm^− 5^ to 822.6 ± 272.4 dyne·s·cm^− 5^ (*P* = 0.025), definitely demonstrating a single BPA session was enough to improve hemodynamics. Except for hemodynamics, PFT indexes, 6MWD, NT-proBNP and WHO-FC all got improved after a single BPA session.

Those receiving multiple BPA sessions got lasting hemodynamic improvements as evidenced by reduced mPAP and PVR together with elevated oxygen saturation before the last BPA session when compared with baseline values. However, CI did not show any significant changes but presented a rising trend after BPA sessions among our patients, while previous studies reported significant improvements in CI [20, 34, 37], which might be ascribed to limited sessions, short BPA intervals and small sample size of our study. Most importantly, LVED got significantly increased, reflecting structural recovery. The above hemodynamic and structural changes are only based on 2–4 BPA sessions, we believe there will be a more significant improvement after more BPA sessions. Furthermore, 6MWD and NT-proBNP got larger scale changes than those in a single BPA session, signifying more benefits brought by staged BPA sessions.

Previous studies reported the rates of lung injury, hemoptysis, and pulmonary artery perforation were 7.0–31.4%, 5.6–19.6%, and 0–8.0% respectively [35]. Occurrence of complications depends on target lesion types, guiding wire types, balloon size and interventionist experience, too many target vessels dilated per session or too large balloon are easy to cause aforementioned complications. No such complications occurred among our patients, we utilized pulmonary angiography and pulmonary perfusion scintigraphy before BPA procedures to facilitate the selection of target lesions, preferred to select smaller balloons (2 mm) at the initial BPA session no matter what lesions our patients had, and limited the total number of balloons (1–2 per session). As subtotal occlusion, total occlusion and tortuous lesions were accompanied with higher risks of complications such as wire injury [38], we started with “webs and bands” and performed balloon dilatation in a staged manner, indicating a relatively conservative approach may lower BPA complications.

CPET is an important clinical tool to evaluate exercise capacity and cardiopulmonary function. Peak VO_2_, an essential factor that mainly reflects exercise capacity and cardiac function, is currently an important index of risk stratification in patients with PAH [39, 40]. In patients with inoperable CTEPH, Peak VO_2_ is still a powerful predictor of mortality [41]. After a median 14.4-week interval between the first and last BPA, Peak VO_2_ and Peak VO_2_% predicted both strikingly increased among our patients receiving multiple BPA sessions, indicating a significantly lasting recovery in exercise capacity and cardiac function and possible improvement of prognosis, other similar parameters such as peak respiratory exchange ratio (respiratory quotient), HRmax and HR% predicted were also comparable to those before BPA, which is consistent with previous studies [20, 34]. VE/VCO_2_ slope, a sensitive variable that reflects exercise ventilatory efficiency, decreased greatly even in the early 1 month after PEA [42] as well as 3 months after BPA sessions [34]. However, VE/VCO_2_ slope showed a non-significantly declining tendency, which might be influenced by small population size.

Unlike earlier studies in which cardiopulmonary function was assessed late after interventions [20, 34], our study mainly focused on the early changes of hemodynamics and cardiopulmonary function within a week after BPA procedure, thus revealing immediate effectiveness of BPA. Peak WR, Peak VO_2_, Peak VO_2_% predicted significantly improved when assessed within 3 days following a single BPA session. OUES is a submaximal exercise parameter that represents the absolute rate of increase in oxygen consumption per 10-fold increase in ventilation, our previous study demonstrated OUES could predict poor outcome in patients with idiopathic PAH [25]. Herein, we observed early increase of OUES after a single BPA session, indicating instant improvement of submaximal exercise capacity. VE and VT are two good indicators of ventilation function, VEmax and VTmax were both evidently elevated, illustrating early ventilation improvements. FVC, FEV1, and MVV have been widely used clinically to evaluate pulmonary function in respiratory diseases, they all achieved instant and lasting correction after BPA procedure, which may be explained by ventilation improvement along with increased pulmonary blood flow so as to maintain the dynamic balance of ventilation and perfusion. In consistent with aforementioned ventilation improvement, CTEPH patients felt much better and breathed more deeper and easier even during BPA procedures in our real-world clinical practice. CPET could objectively, safely and repetitiously evaluate the cardiopulmonary function, exhibiting absolute advantages over subjective parameters such as WHO-FC and 6MWD as well as catherization hemodynamics which might be confounded by supplemental oxygen during BPA, CPET may exert as a sensitive and reliable indicator to assess the effects of BPA.

There are several limitations of this study. First, the sample size of this study is relatively small. Second, this study was implemented in a single center, although our center is the largest pulmonary vascular center and also one of the largest BPA centers in China. Large-scale, multicenter, prospective studies should be launched to investigate and confirm the long-term effects of BPA procedures for CTEPH patients. Third, we didn’t measure other parameters such as oxygen saturation after BPA for economic issues and procedure time, thus immediate PVR and CI were not obtained, leading to incomprehensive hemodynamics evaluation after a single BPA session.

## Conclusion

BPA can safely and effectively improve the instant and lasting hemodynamics and cardiopulmonary function. Larger multicenter studies are needed to further investigate and confirm the role of BPA in CTEPH.

## Supplementary information

**Additional file 1.** Representative videos before and immediately after BPA.

**Additional file 2: Figure S1.** Hemodynamic and clinical parameters before and after multiple BPA sessions. **Figure S2.** CPET parameters before and after multiple BPA sessions.

## Data Availability

All data generated or analyzed during this study are included in this manuscript.

## Abbreviations

6MWD: 6-min walk distance
AT: Anaerobic threshold
BPA: Balloon pulmonary angioplasty
CI: Cardiac index
CPET: Cardiopulmonary exercise testing
CTEPH: Chronic thromboembolic pulmonary hypertension
DVT: Deep vein thrombosis
FEV1: Forced expiratory volume in the first second
FVC: Forced vital capacity
LVED: Left ventricular end-diastolic diameter
mPAP: Mean pulmonary arterial pressure
MVV: Maximal voluntary ventilation
NT-proBNP: N-terminal pro-brain natriuretic peptide
OUES: Oxygen uptake efficiency slope
PAH: Pulmonary arterial hypertension.
PAWP: Pulmonary arterial wedge pressure
PEA: Pulmonary endarterectomy
PH: Pulmonary hypertension
PVR: Pulmonary vascular resistance
RAP: Right atrium pressure
RHC: Right heart catheterization
RVED: Right ventricular end-diastolic diameter
SaO2: Oxygen saturation
SvO2: Mixed venous oxygen saturation
TAPSE: Tricuspid annular plane systolic excursion
TTE: Transthoracic echocardiography
VE: Minute ventilation
WHO-FC: World Health Organization functional class
WR: Work rate
