# Supplementary figures and images for: Improved hemodynamics and cardiopulmonary function in patients with inoperable chronic thromboembolic pulmonary hypertension after balloon pulmonary angioplasty
Source: Respir Res. 2019 Nov 8;20:250. doi: 10.1186/s12931-019-1211-y (PMC6842206; doi:10.1186/s12931-019-1211-y)

## Slide 1
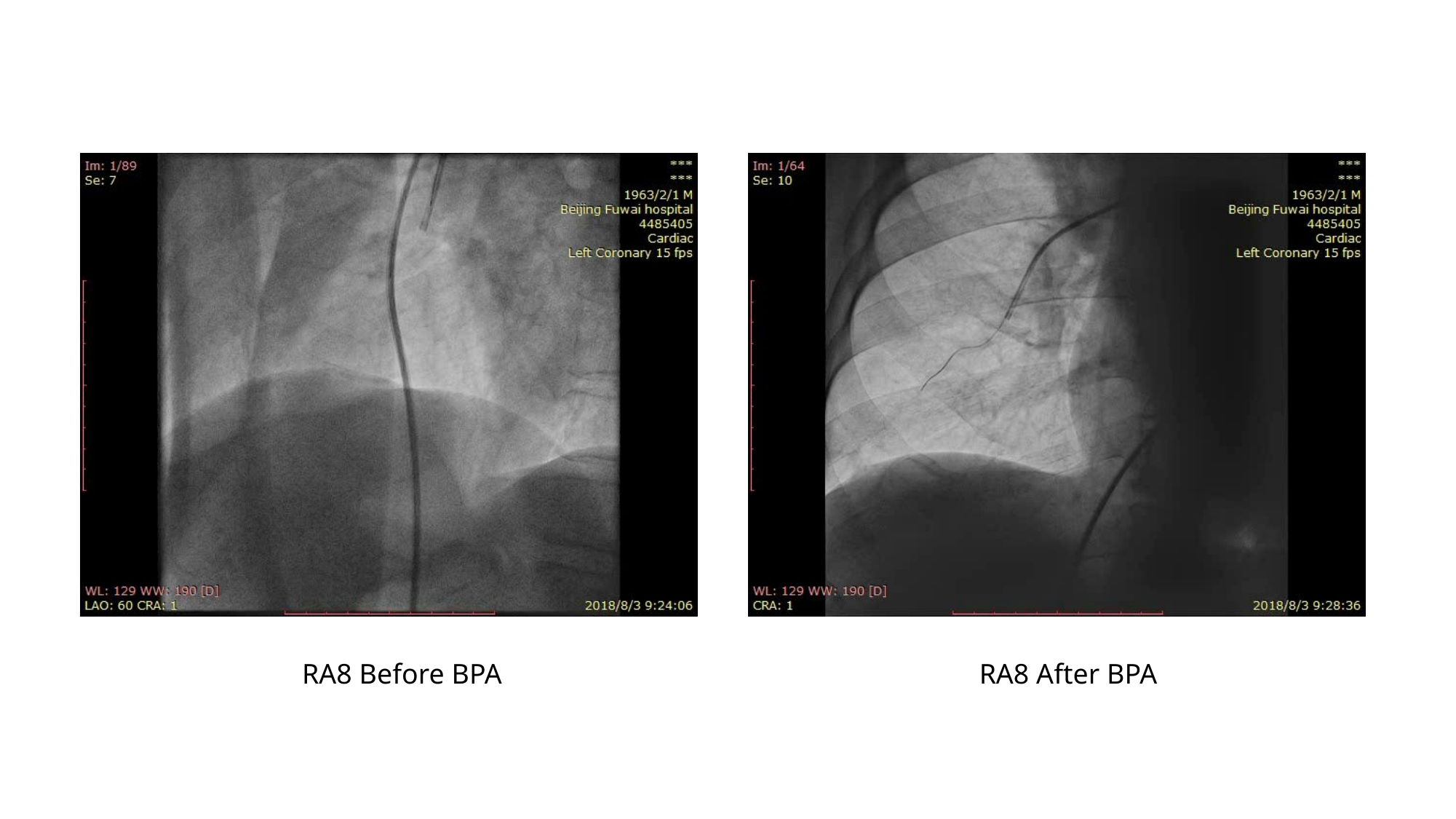

RA8 Before BPA
RA8 After BPA

## Slide 2
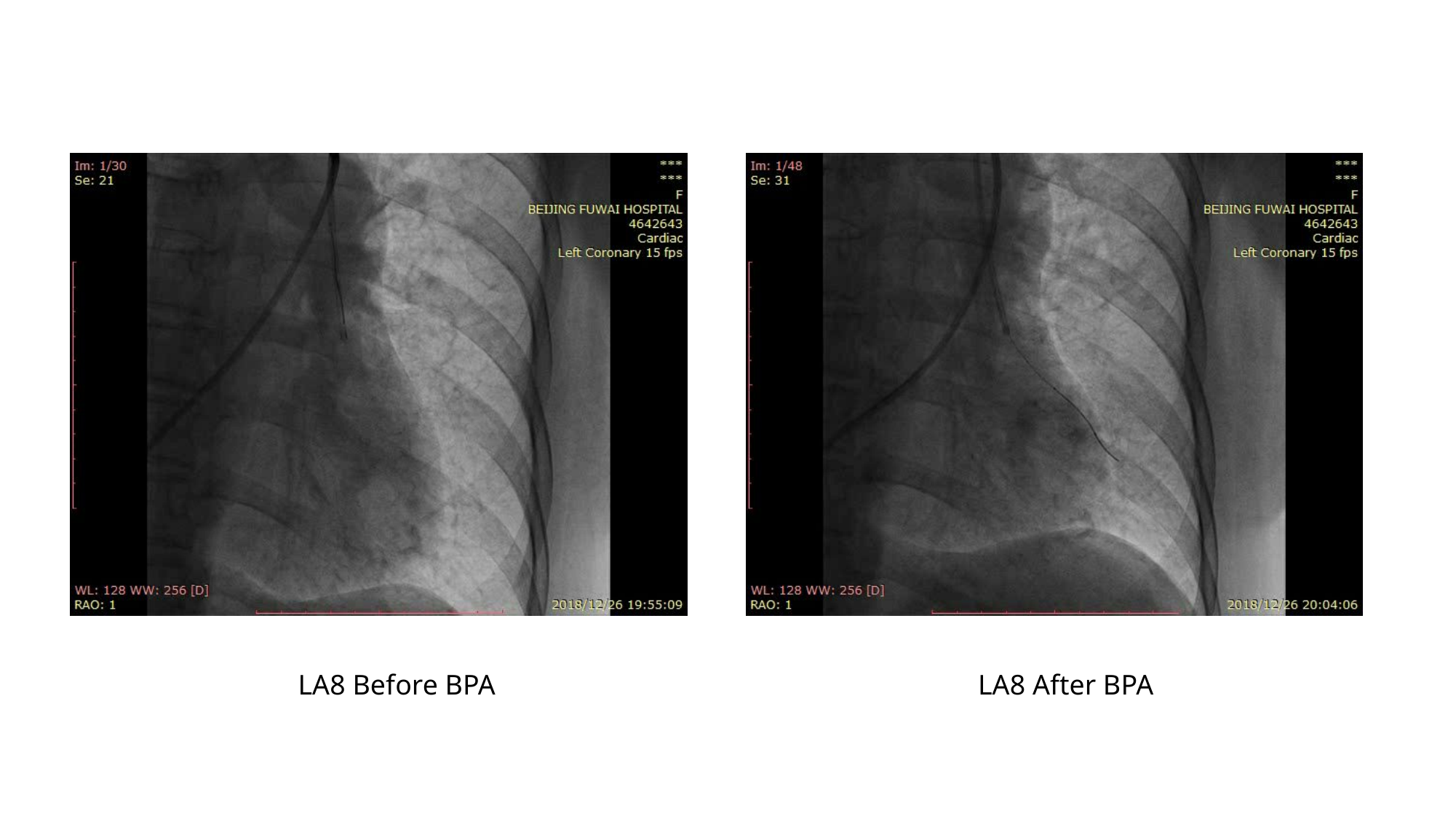

LA8 Before BPA
LA8 After BPA

## Slide 3
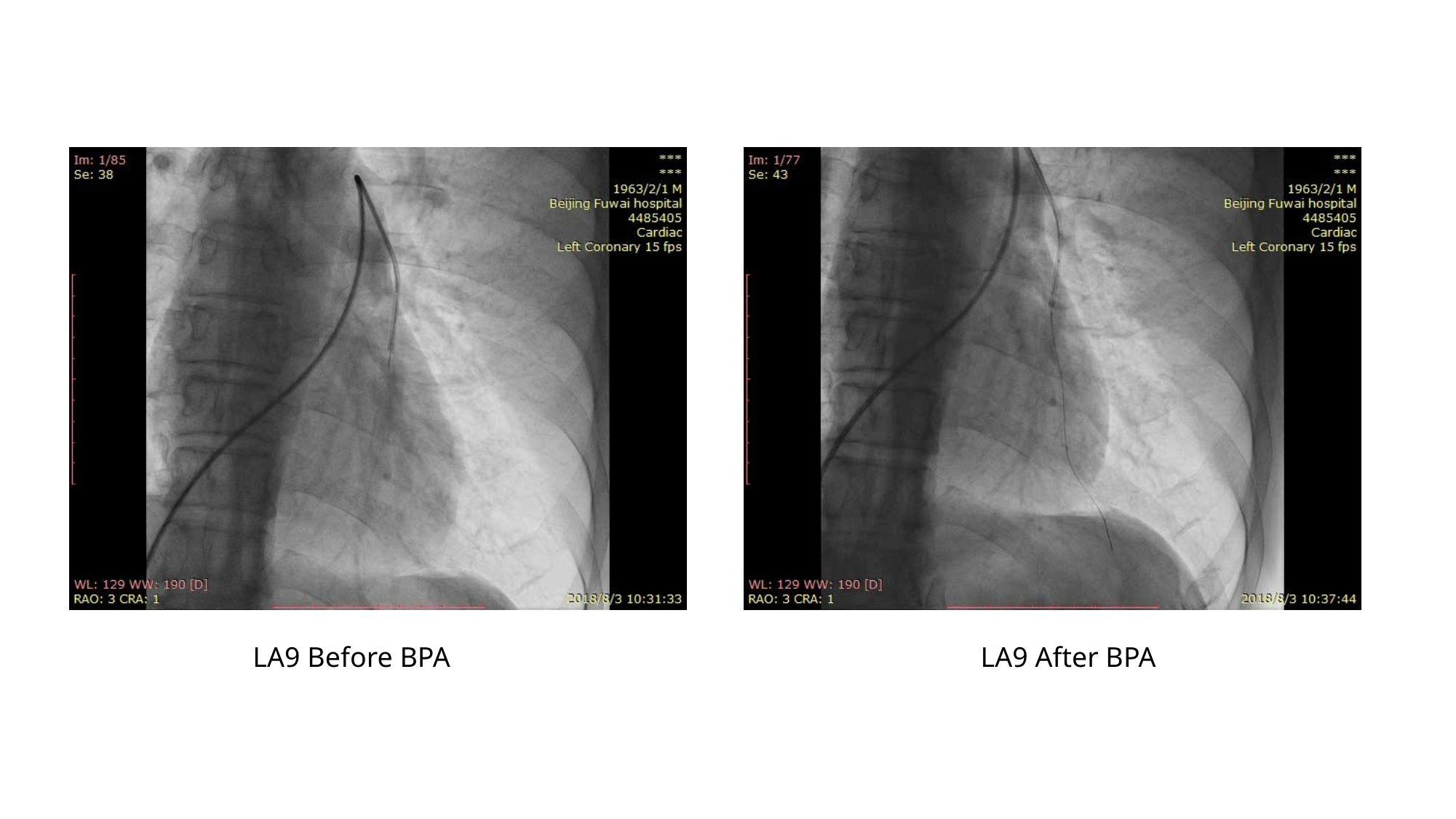

LA9 Before BPA
LA9 After BPA

Supplement: Supplementary file 1 — Additional file 1. Representative videos before and immediately after BPA. [file 12931_2019_1211_MOESM1_ESM.pptx]
