# Supplementary material for: Improved hemodynamics and cardiopulmonary function in patients with inoperable chronic thromboembolic pulmonary hypertension after balloon pulmonary angioplasty
Source: Respir Res. 2019 Nov 8;20:250. doi: 10.1186/s12931-019-1211-y (PMC6842206; doi:10.1186/s12931-019-1211-y)
Supplement: Supplementary file 2 — Additional file 2: Figure S1. Hemodynamic and clinical parameters before and after multiple BPA sessions. Figure S2. CPET parameters before and after multiple BPA sessions. [file 12931_2019_1211_MOESM2_ESM.docx]

**Figure S1.** Hemodynamic and clinical parameters before and after multiple BPA sessions





**Figure S2**. CPET parameters before and after multiple BPA sessions.
